# Foliar Application of Amino Acids Increases *Sweet Basil* (*Ocimum basilicum* L.) Resistance to High-Temperature Stress

**DOI:** 10.3390/plants14050739

**Published:** 2025-02-28

**Authors:** Justina Deveikytė, Aušra Blinstrubienė, Natalija Burbulis, Aldona Baltušnikienė

**Affiliations:** 1 Department of Plant Biology and Food Sciences, Vytautas Magnus University, Donelaicio Str. 58, 44248 Kaunas, Lithuania; ausra.blinstrubiene@vdu.lt; 2 Bioeconomy Research Institute, Vytautas Magnus University, Donelaicio Str. 58, 44248 Kaunas, Lithuania; natalija.burbulis@vdu.lt; 3 Department of Biochemistry, Faculty of Medicine, Lithuanian University of Health Sciences, Mickeviciaus Str. 9, 44307 Kaunas, Lithuania; aldona.baltusnikiene@lsmuni.lt

**Keywords:** abiotic stress, amino acid, antioxidant activity, chlorophyll, total phenolic content

## Abstract

Climate change poses a significant threat to food security, with projections indicating a decline in crop yield due to reduced crop growth in the face of rising temperatures. This study evaluated the effects of L-Isoleucine, L-Methionine, L-Glutamine, L-Tryptophan, and L-Phenylalanine on the morphophysiological parameters, total phenolic content, and antioxidant activity of *Sweet Basil* (*Ocimum basilicum* L.) under high-temperature stress. Ten cultivar varieties of the sweet basil, “Rosie”, “Red Opal”, “Bordeaux”, “Dark Opal”, “Red Rubin”, “Genovese”, “Cinamon”, “Italiano Classico”, “Marseillais”, and “Thai”, were grown in a controlled-environment growth chamber. The seedlings with 5–6 true leaves were divided into seven groups: the first group of seedlings had no treatment and was grown under 25/22 °C (day/night) temperature, the second group of seedlings had no treatment and was grown under 35/30 °C (day/night) temperature, and the remaining five groups were sprayed with 100 mg L^−1^ of L-Isoleucine, L-Methionine, L-Glutamine, L-Tryptophan, or L-Phenylalanine. As our results show, L-Tryptophan increased fresh and dry biomass in green sweet basil, while L-Methionine had the greatest effect on biomass in purple varieties. L-Phenylalanine increased chlorophyll *a* and *b* in heat-stressed “Bordeaux” (purple variety) and “Marseillais” (green variety). L-Isoleucine and L-Glutamine increased total phenolic compounds (TPCs) in purple cultivars (“Rosie”, “Red Opal”, “Dark Opal”, and “Red Rubin”), while L-Tryptophan (“Cinamon” and “Italiano Classico”) and L-Phenylalanine increased TPCs in “Cinamon”, “Marseillais”, and “Thai” green cultivars. Antioxidant activity (ABTS) was highest in “Dark Opal” and “Bordeaux” treated with L-Tryptophan or L-Phenylalanine under heat stress, while “Thai” benefited most from L-Isoleucine. The exogenous application of amino acids could serve as a viable solution to alleviate the negative effects of temperature stress on sweet basil and serve as an environmentally friendly agricultural strategy.

## 1. Introduction

*Sweet basil* (*Ocimum basilicum* L.) is an aromatic herb of the “Lamiaceae” family, valued for its culinary, medicinal, and antimicrobial properties. Its essential oil contains eugenol, methyl chavicol, and linalool, contributing to its antioxidant, antibacterial, and antifungal properties [1,2,3]. The composition of the essential oil strongly varies with environmental and cultivation conditions, impacting its use in food and pharmaceuticals [1,4]. Basil offers health benefits such as blood sugar regulation, anti-inflammatory effects, and immune support [5,6]. Rich in polyphenols like quercetin and kaempferol, it serves as an antioxidant and natural preservative [5,7]. *Sweet basil’s* antimicrobial properties help extend food shelf life by inhibiting pathogens [8,9]. Beyond culinary uses, basil oil is valued in cosmetics and pharmaceuticals, particularly for insect repellent and antimicrobial applications [2,8]. Its economic significance spans food, cosmetics, and herbal medicine, making it a valuable crop worldwide [10,11,12].

*Sweet basil* is typically associated with warmer climates, with an optimal temperature range for its growth between 25 °C and 30 °C [13]. However, it has been observed that climate change and sudden temperature fluctuations can have a detrimental effect on the growth and development of these plants [14]. With population growth and industrial development on the rise, global warming has become an undeniable challenge [15,16]. The growth and development of crops are strongly influenced by environmental temperature, making it one of the most important factors [17]. Elevated temperature stress is regarded as a result of recent climate changes, potentially leading to a significant reduction in the yield of various crops and vegetables in the coming years [14,18,19]. It is predicted that, in the absence of the benefits provided by CO_2_ fertilization, effective adaptation strategies, and genetic improvement, a 1 °C increase in global mean temperature could result in a reduction in global yields from 3.1% to 7.4% for a variety of plants (soybean, rice, wheat, and maize) [20]. Temperature is one of the critical factors affecting the photosynthesis, physiological functions, and developmental stages of many crops, especially C3 crops. It affects enzyme activity and metabolic rates; hence, high-temperature stress results in decreasing growth, quality, and plant yield [14,21].

In challenging growth conditions (e.g., elevated temperatures), many plants, including *Sweet Basil*, have evolved to adapt their metabolism in order to reduce negative effects and survive unfavorable abiotic or biotic factors [19,22,23]. The synthesis of secondary metabolites in *Sweet Basil* contributes to adapting to environmental conditions as a protective response to external stimuli [24]. To mitigate these stresses, plants engage different non-enzymatic antioxidants like phenolics, flavonoids, carotenoids, and glucosinolates, which function as a non-enzymatic defense system against biotic and abiotic stressors. These well-studied secondary metabolites hold significant value for their applications, prompting initiatives to boost their biosynthesis pathways and increase their levels [25,26,27].

To further enhance the resilience of *Sweet Basil* to high-temperature stress, the application of certain substances, such as amino acids, has been proposed as an effective agronomic strategy. Amino acids, when used as foliar sprays, can stimulate plant growth and improve quality by optimizing nutrient uptake, play vital roles in plant metabolism, improving growth and vitamin biosynthesis, and enhance tolerance to various environmental stresses, including drought, salinity, and high-temperature stress [28,29].

Biostimulants, which include amino acids, enhance plant growth, stress resistance, soil fertility, and nutrient use efficiency. Amino acids L-Phenylalanine and L-Tryptophan are precursors to various secondary metabolites, including flavonoids and phenolic compounds, which are known to protect plants from oxidative damage during thermal stress. The accumulation of these metabolites can enhance the plant’s antioxidant capacity, thereby mitigating the effects of reactive oxygen species (ROS) generated under high-temperature conditions [30]. Additionally, L-Phenylalanine is involved in the synthesis of phytoalexins, which are antimicrobial compounds that can also contribute to stress resilience [31]. L-Glutamine is crucial for nitrogen metabolism and can act as a nitrogen donor in various biosynthetic pathways. Under high-temperature stress, glutamine can help maintain cellular homeostasis by participating in the synthesis of other amino acids and proteins that are essential for stress response [32]. Furthermore, glutamine has been shown to play a role in the regulation of osmotic balance, which is vital for plant survival under heat-stress conditions [32]. L-Methionine is particularly noteworthy for its role in enhancing plant stress tolerance. It serves as a precursor for S-adenosylmethionine (SAM), which is involved in ethylene production—a hormone that regulates plant responses to stress. Methionine also contributes to the synthesis of glutathione, a key antioxidant that helps scavenge ROS and reduce oxidative damage during heat stress [33]. Studies have indicated that exogenous application of methionine can improve the antioxidant capacity of plants, thereby enhancing their ability to withstand high temperatures [34,35]. Branched-chain amino acid L-Isoleucine has been implicated in stress signaling pathways. It is known to modulate the production of stress-related hormones and can enhance the synthesis of protective proteins under heat-stress conditions. L-Isoleucine also plays a role in the regulation of metabolic pathways that are crucial for maintaining cellular integrity during thermal stress [36].

The amino acids L-Phenylalanine (2-amino-3-phenylpropanoic acid), L-Tryptophan (2-amino-3-(1H-indol-3-yl)propanoic acid), L-Glutamine (2-amino-4-carbamoylbutanoic acid), L-Methionine (2-amino-4-(methylthio)butanoic acid), and L-Isoleucine (2-amino-3-methylpentanoic acid) are key players in plant responses to high-temperature stress. The present study was conducted with the hypothesis that exogenously applied amino acids can mitigate the adverse effects of high temperatures. To test this hypothesis, the effects of L-Isoleucine, L-Methionine, L-Glutamine, L-Tryptophan, and L-Phenylalanine on the morphophysiological parameters, total phenolic content, and antioxidant activity *Sweet Basil* under high-temperature conditions were investigated.

## 2. Results

### 2.1. The Impact of High-Temperature Stress and Amino Acid Supplementation on the Fresh and Dry Weights

**Figure 1:**
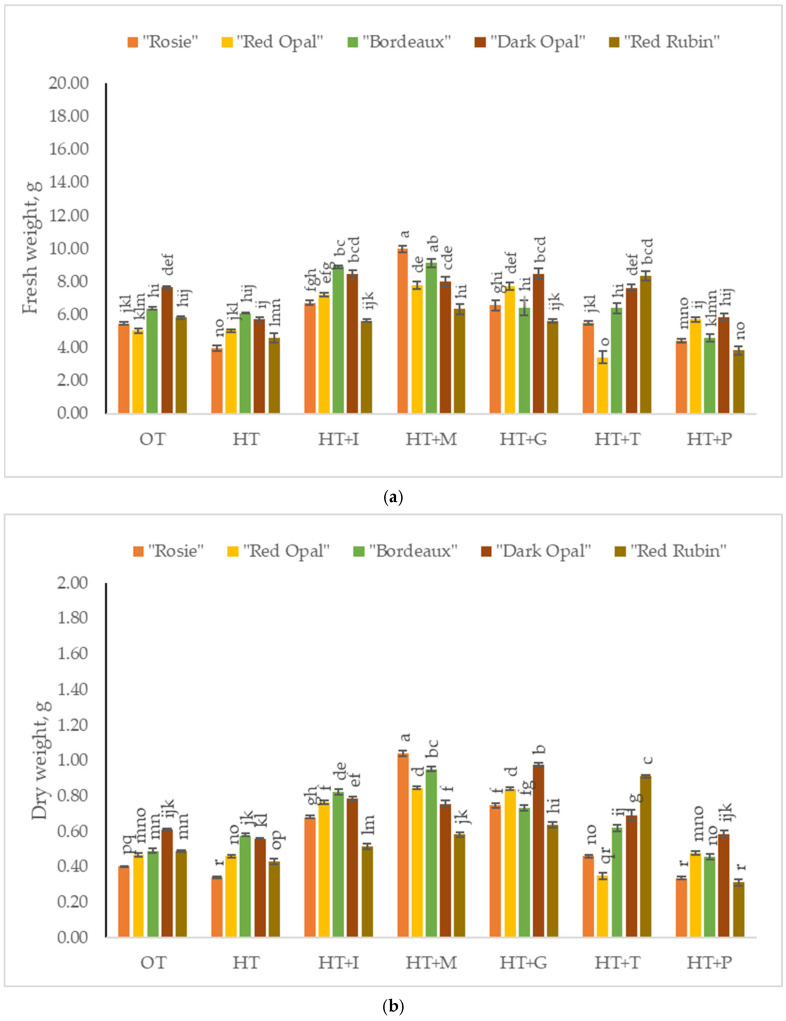
(**a**). Fresh weight of *Sweet Basil* (*Ocimum basilicum* L.) purple varieties grown at optimum and high temperatures and sprayed with amino acids. Data are presented as mean ± standard error. Means sharing a different letter are significantly different at the *p* < 0.05 level. (**b**). Dry weight of *Sweet Basil* purple varieties grown at optimum and high temperatures and sprayed with amino acids. Data are presented as mean ± standard error. Means sharing a different letter are significantly different at the *p* < 0.05 level. Note: OT—Optimal temperature; HT—High-temperature stress; HT+I—High-temperature stress + L-Isoleucine; HT+M—High-temperature stress + L-Methionine; HT+G—High-temperature stress + L-Glutamine; HT+T—High-temperature stress + L-Tryptophan; HT+P—High-temperature stress + L-Phenylalanine.

The fresh weight of *Sweet Basil* plants grown under optimal temperature conditions varied from 5.02 g to 7.64 g (Figure 1a), and the dry weight varied from 0.40 g to 0.61 g (Figure 1b) depending on the purple varieties’ cultivars. Foliar application of L-Methionine resulted in the highest fresh and dry weights of varieties “Rosie”, “Red Opal”, and “Bordeaux”, while the highest fresh and dry weights of “Dark Opal” have been obtained under the application of L-Glutamine. L-Tryptophan significantly increased the fresh and dry weights of “Red Rubin”.

**Figure 2:**
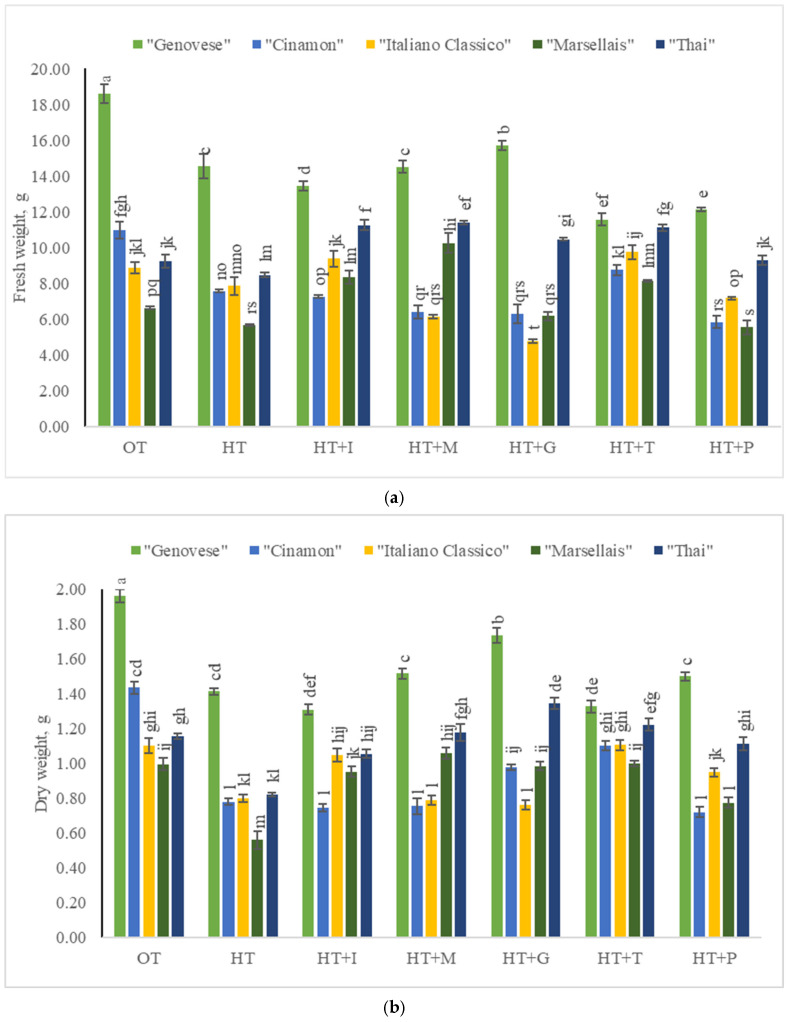
(**a**). Fresh weight of *Sweet Basil* green varieties grown at optimum and high temperatures and sprayed with amino acids. Data are presented as mean ± standard error. Means sharing a different letter are significantly different at the *p* < 0.05 level. (**b**). Dry weight of *Sweet Basil* green varieties grown at optimum and high temperatures and sprayed with amino acids. Data are presented as mean ± standard error. Means sharing a different letter are significantly different at the *p* < 0.05 level. Note: OT—Optimal temperature; HT—High-temperature stress; HT+I—High-temperature stress + L-Isoleucine; HT+M—High-temperature stress + L-Methionine; HT+G—High-temperature stress + L-Glutamine; HT+T—High-temperature stress + L-Tryptophan; HT+P—High-temperature stress + L-Phenylalanine.

Depending on the cultivar of the green variety, the fresh weight of *Sweet Basil* plants grown under optimal temperature varied from 6.63 g to 18.60 g (Figure 2a), while the dry weight varied from 1.00 g to 1.96 g (Figure 2b). Exogenously applied L-Methionine resulted in increasing fresh and dry weights of varieties “Marseillais” and “Thai”, while L-Tryptophan significantly increased the fresh and dry weights of cultivars “Cinamon” and “Italiano Classico”.

### 2.2. The Impact of High-Temperature Stress and Amino Acid Supplementation on Chlorophyll a and b Content

**Figure 3:**
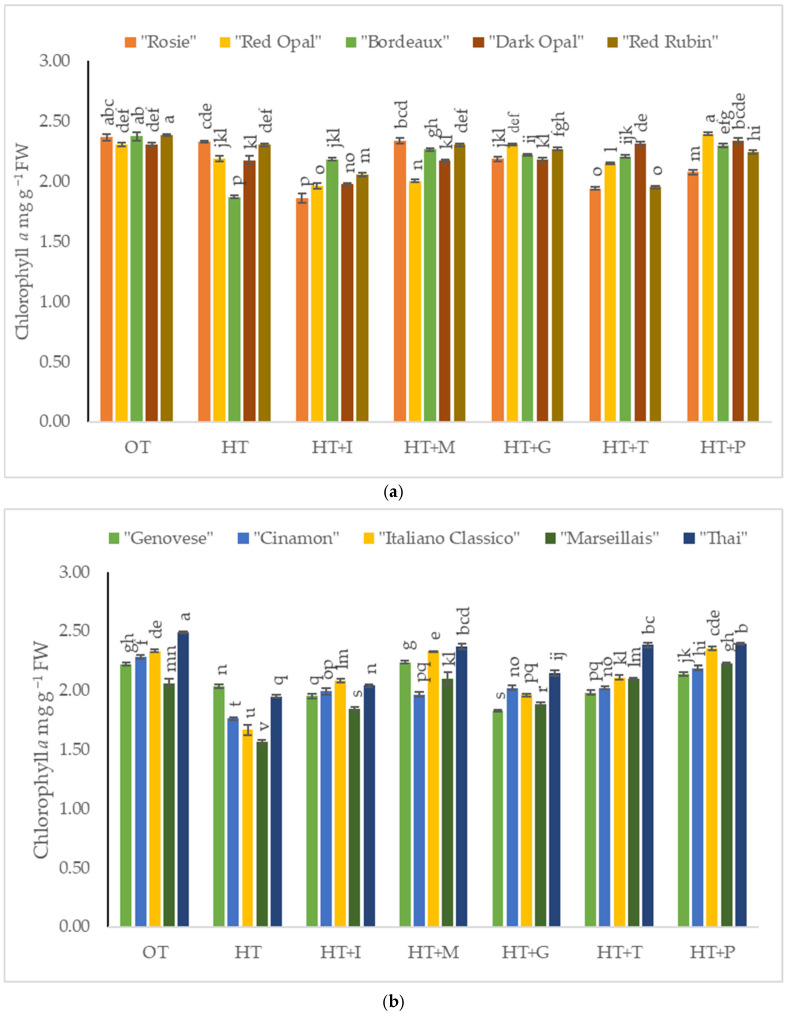
(**a**). Chlorophyll *a* content in *Sweet Basil* purple varieties grown at optimum and high temperatures and sprayed with amino acids. Data are presented as mean ± standard error. Means sharing a different letter are significantly different at the *p* < 0.05 level. (**b**). Chlorophyll *a* content in *Sweet Basil* green varieties grown at optimum and high temperatures and sprayed with amino acids. Data are presented as mean ± standard error. Means sharing a different letter are significantly different at the *p* < 0.05 level. Note: OT—Optimal temperature; HT—High-temperature stress; HT+I—High-temperature stress + L-Isoleucine; HT+M—High-temperature stress + L-Methionine; HT+G—High-temperature stress + L-Glutamine; HT+T—High-temperature stress + L-Tryptophan; HT+P—High-temperature stress + L-Phenylalanine.

The chlorophyll *a* content in *Sweet Basil* plants grown under optimal temperature conditions varied from 2.30 mg g^−1^ FW to 2.38 mg g^−1^ FW depending on the purple varieties’ cultivars (Figure 3a) and from 2.06 mg g^−1^ FW to 2.49 mg g^−1^ FW depending on the green varieties’ cultivars (Figure 3b). High-temperature stress significantly decreased chlorophyll *a* content in purple varieties’ cultivars “Red Opal”, “Bordeaux”, “Dark Opal”, and “Red Rubin”, while it had no effect in the “Rosie” cultivar (Figure 3a). The application of L-Methionine significantly increased chlorophyll *a* content in purple varieties’ cultivars “Rosie” and “Red Rubin” and in green varieties’ “Genovese”, while in cultivars “Red Opal”, “Bordeaux”, “Dark Opal”, “Cinamon”, “Italiano Classico”, “Marseillais”, and “Thai”, chlorophyll *a* content was significantly increased by the application of L-Phenylalanine.

**Figure 4:**
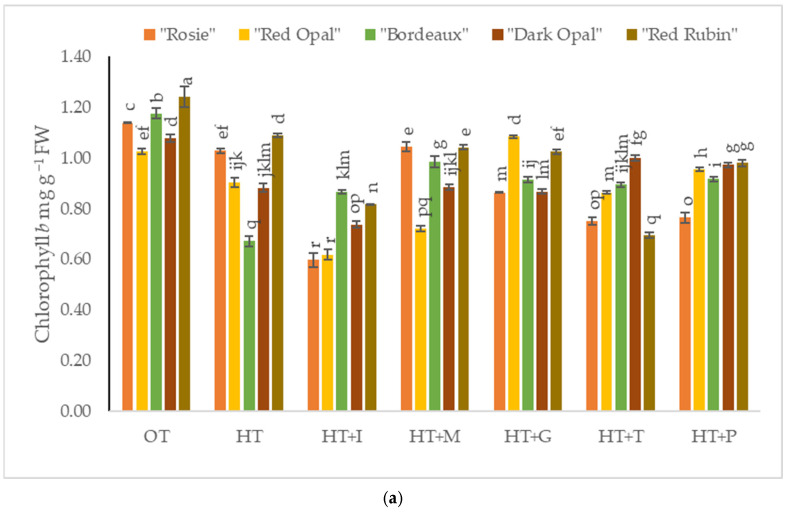

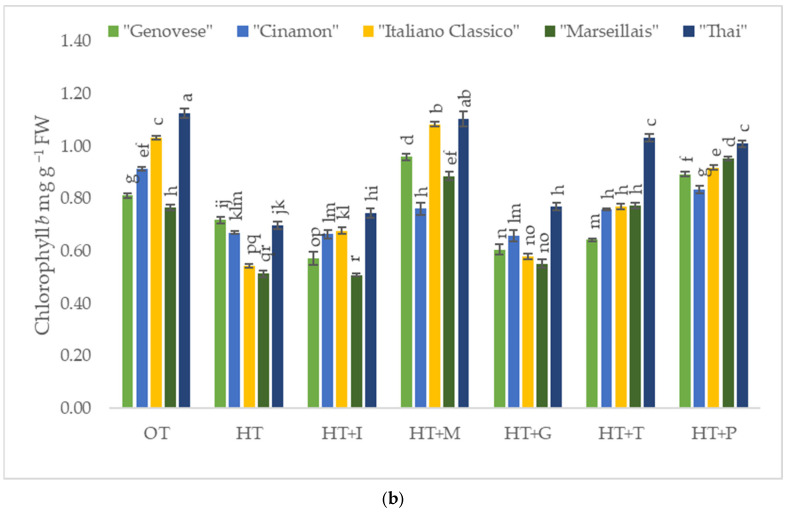
(**a**). Chlorophyll *b* content in *Sweet Basil* purple varieties grown at optimum and high temperatures and sprayed with amino acids. Data are presented as mean ± standard error. Means sharing a different letter are significantly different at the *p* < 0.05 level. (**b**). Chlorophyll *b* content in *Sweet Basil* green varieties grown at optimum and high temperatures and sprayed with amino acids. Data are presented as mean ± standard error. Means sharing a different letter are significantly different at the *p* < 0.05 level. Note: OT—Optimal temperature; HT—High-temperature stress; HT+I—High-temperature stress + L-Isoleucine; HT+M—High-temperature stress + L-Methionine; HT+G—High-temperature stress + L-Glutamine; HT+T—High-temperature stress + L-Tryptophan; HT+P—High-temperature stress + L-Phenylalanine.

The chlorophyll *b* content in *Sweet Basil* plants grown under optimal temperature conditions varied from 1.03 mg g^−1^ FW to 1.24 mg g^−1^ FW depending on the purple varieties’ cultivars (Figure 4a) and from 0.77 mg g^−1^ FW to 1.13 mg g^−1^ FW depending on the green varieties’ cultivars (Figure 4b). High-temperature stress significantly decreased chlorophyll *b* content in the purple varieties’ cultivars compared to *Sweet Basil* plants grown under optimal temperature conditions. Foliar application of L-Methionine to high-temperature stressed plants increased chlorophyll *b* content in the cultivars “Genovese” and “Italiano Classico”, while the highest chlorophyll *b* content of “Red Opal” was obtained under the application of L-Glutamine. The foliar spraying with L-Phenylalanine had a positive effect on the green variety cultivar “Marseillais”.

### 2.3. The Impact of High-Temperature Stress and Amino Acid Supplementation on the Total Phenolic Content

**Figure 5:**
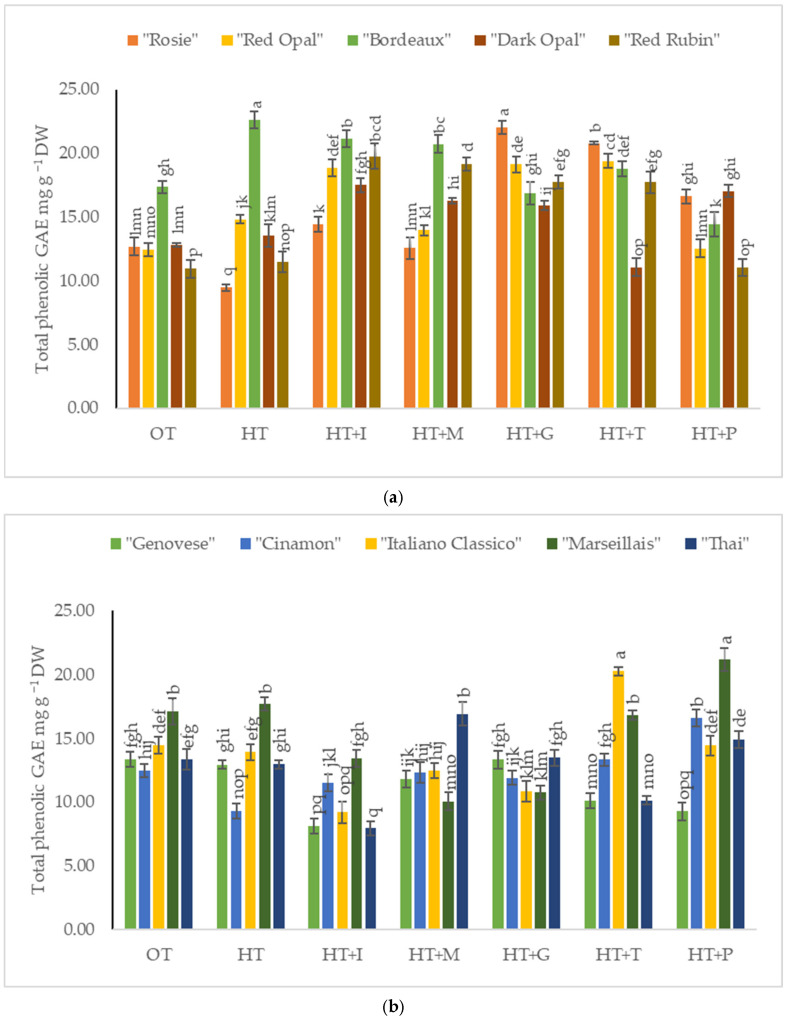
(**a**). Total phenolic content in *Sweet Basil* purple varieties grown at optimum and high temperatures and sprayed with amino acids. Data are presented as mean ± standard error. Means sharing a different letter are significantly different at the *p* < 0.05 level. (**b**). Total phenolic content in *Sweet Basil* green varieties grown at optimum and high temperatures and sprayed with amino acids. Data are presented as mean ± standard error. Means sharing a different letter are significantly different at the *p* < 0.05 level. Note: OT—Optimal temperature; HT—High-temperature stress; HT+I—High-temperature stress + L-Isoleucine; HT+M—High-temperature stress + L-Methionine; HT+G—High-temperature stress + L-Glutamine; HT+T—High-temperature stress + L-Tryptophan; HT+P—High-temperature stress + L-Phenylalanine.

The total phenolic content (TPC) in *Sweet Basil* plants grown under optimal temperature conditions varied from 10.94 mg g^−1^ DW to 17.36 mg g^−1^ DW depending on the purple varieties’ cultivars (Figure 5a) and from 12.47 mg g^−1^ DW to 17.12 mg g^−1^ DW depending on the green varieties’ cultivars (Figure 5b). High-temperature stress significantly increased the total phenolic content in the purple varieties’ cultivars “Bordeaux” and “Red Opal” compared to *Sweet Basil* plants grown under optimal temperature conditions, decreased the TPC in “Rosie”, and had no effect on other cultivars tested. High-temperature stress decreased the total phenolic content in the green varieties’ cultivar “Cinamon” compared to *Sweet Basil* plants grown under optimal temperature conditions and had no effect on other cultivars tested. Foliar application of L-Isoleucine resulted in the highest total phenolic content in the varieties “Dark Opal” and “Red Rubin”. A significant increase in the total phenolic content under the application of L-Methionine was observed for the cultivar “Thai”, while the TPCs in the varieties “Red Opal” and “Italiano Clasico” were obtained under the application of L-Tryptophan. Exogenously applied L-Phenylalanine significantly increased the TPCs of varieties “Cinamon” and “Marseillais”.

### 2.4. The Impact of High-Temperature Stress and Amino Acid Supplementation on the Antioxidant Activity

**Figure 6:**
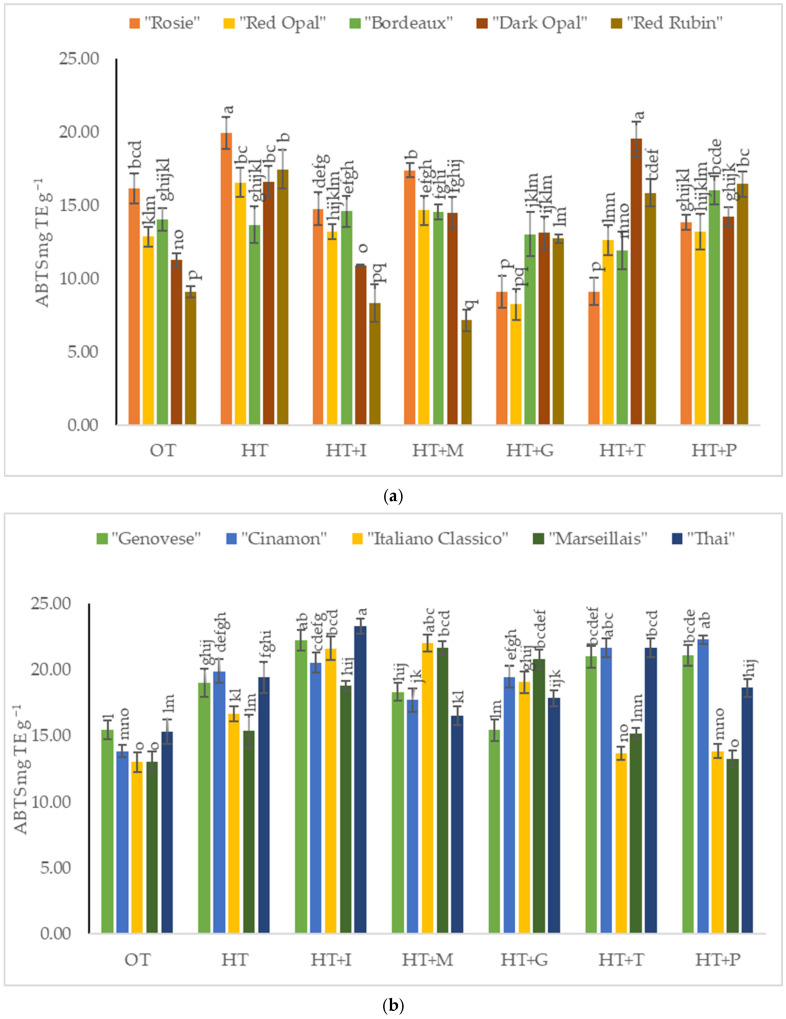
(**a**). Antioxidant activity of basil leaf extracts determined by ABTS assay content in *Sweet Basil* purple varieties grown at optimum and high temperatures and sprayed with amino acids. Data are presented as mean ± standard error. Means sharing a different letter are significantly different at the *p* < 0.05 level. (**b**). Antioxidant activity of basil leaf extracts determined by ABTS assay content in *Sweet Basil* green varieties grown at optimum and high temperatures and sprayed with amino acids. Data are presented as mean ± standard error. Means sharing a different letter are significantly different at the *p* < 0.05 level. Note: OT—Optimal temperature; HT—High-temperature stress; HT+I—High-temperature stress + L-Isoleucine; HT+M—High-temperature stress + L-Methionine; HT+G—High-temperature stress + L-Glutamine; HT+T—High-temperature stress + L-Tryptophan; HT+P—High-temperature stress + L-Phenylalanine.

The antioxidant activity measured by the ABTS method in *Sweet Basil* plants grown under optimal temperature conditions varied from 9.11% to 16.16% depending on the cultivars of purple varieties (Figure 6a) and from 13.03% to 15.49% depending on the cultivars of green varieties (Figure 6b). High-temperature stress significantly increased antioxidant activity in the purple varieties’ cultivars compared to *Sweet Basil* plants grown under optimal temperature conditions, except for the cultivar “Bordeaux” and all the green varieties’ cultivars tested. Foliar application of L-Isoleucine resulted in a significant increase in antioxidant activity in the cultivars “Genovese” and “Thai”, while the highest antioxidant activity of “Italiano Classico” and “Marseillais” was obtained under the application of L-Methionine. Significantly increased antioxidant activity was determined under the application of L-Tryptophan for the cultivar “Dark Opal”. The foliar spraying with L-Phenylalanine resulted in increased antioxidant activity in the cultivars “Bordeaux” and “Cinamon”.

## 3. Discussion

High-temperature stress is a critical factor that negatively impacts the growth and development of *Sweet Basil*, leading to significant reductions in crop growth rates, chlorophyll content, photosynthesis, and biomass accumulation. The physiological responses of *Sweet Basil* to elevated temperatures are complex and involve various biochemical pathways that ultimately affect plant health and productivity. Scientific research indicates that nearly all purple basil cultivars have less fresh weight and fewer nodes than their green counterparts. The reduced growth was attributed to elevated levels of anthocyanins in purple basil. These compounds are typically associated with the inhibition of growth in comparison to similar green-leaved varieties [37]. Prinsi et al. reported that the cultivation of green basil (*O. basilicum* var. “Italiano Classico”) resulted in a greater accumulation of biomass than that of the purple variety (*O. basilicum* var. “Red Rubin”) [38]. The significant impact of the foliar application of L-Methionine on biomass accumulation in purple basil can be attributed to its multifaceted role in enhancing photosynthesis and improving nutrient uptake in lettuce, safflower, and chickpea [34,39,40]. The differential responses of green and purple basil varieties to L-Tryptophan and L-Methionine can be explained by their unique metabolic pathways and physiological characteristics. Green basil benefits from L-Tryptophan’s role in auxin synthesis, leading to enhanced biomass, while purple basil’s response to L-Methionine is linked to its metabolic pathways that favor anthocyanin production, which may influence growth dynamics in a different manner.

The chlorophyll content in *Sweet Basil* varies between green and purple varieties, influenced by physiological and environmental factors. Research indicates that purple basil generally exhibits higher chlorophyll content compared to green varieties. For instance, Hendrickson et al. reported that SPAD readings, which are indicative of chlorophyll content, were higher in purple basil than in most green cultivars [41]. Eskandarzade et al. noted that purple basil accumulates more chlorophyll, particularly chlorophyll *b*, as a response to the influence of negative abiotic factors [42]. Our investigation also revealed that the highest chlorophyll *b* content was determined in the purple varieties’ *Sweet Basil* cultivar “Red Rubin”, while the highest chlorophyll *a* content was determined in the green varieties’ *Sweet Basil* cultivar “Thai”. It is known that elevated temperature can significantly impair chlorophyll synthesis and stability in plants. Barickman et al. found that temperature stress negatively affected the chlorophyll content in basil, leading to a reduction in chlorophyll levels and impairing the xanthophyll cycle pigments, which are essential for photosynthetic efficiency [14]. Reduction in chlorophyll under unfavorable growth conditions is often associated with increased oxidative stress, which can be alleviated by the application of certain amino acids [43,44,45]. The results of our study have shown that the application of L-Phenylalanine to high-temperature stressed plants resulted in a significant increase in chlorophyll *a* content in the purple variety “Bordeaux” and in the green variety *Sweet Basil* “Marseillais”. Furthermore, the same amino acid significantly increased the chlorophyll *b* content by 86% in the green variety “Marseillais” compared to high-temperature stressed plants, and the application of L-Methionine to high-temperature stressed plants substantially elevated the chlorophyll *b* content in the purple variety “Bordeaux”. L-Phenylalanine has been shown to enhance the overall antioxidant capacity of plants, thereby improving their ability to cope with oxidative damage caused by temperature stress. Its application has been linked to improved chlorophyll retention under stress conditions because phenylalanine may activate chlorophyllase, an enzyme involved in chlorophyll metabolism, thereby enhancing chlorophyll synthesis and reducing degradation [14,46,47]. Similarly, L-Methionine has been shown to enhance the synthesis of ethylene, which can regulate chlorophyll degradation and promote chloroplast stability under high-temperature conditions [14,19,34].

Research studies have reported that the synthesis of phenolic compounds is often enhanced under stress conditions, as these compounds play a role in protecting plants from environmental stressors [48,49]. Amino acids have been shown to play a significant role in enhancing the synthesis of phenolic compounds in various plant species. The application of amino acids could potentially mitigate the negative effects of high temperatures on *Sweet Basil* by promoting the synthesis of phenolic compounds, thus enhancing the plant’s antioxidant capacity [48]. This relationship highlights the potential for using amino acid treatments as a strategy to enhance the phenolic content of *Sweet Basil* under high-temperature stress. In our study, it was determined that L-Isoleucine and L-Glutamine had a significant influence on the increase in phenolic compounds in the purple varieties’ cultivars “Rosie”, “Red Opal”, “Dark Opal”, and “Red Rubin”, while L-Tryptophan (“Cinamon” and “Italiano Classico”) and L-Phenylalanine (“Cinamon”, “Marseillais”, and “Thai”) affected certain green varieties’ cultivars. The differences in antioxidant activity between green and purple basil varieties may be influenced by factors like the accumulation of other phenolic compounds (rosmarinic acid and chicoric acid) [38,50]. Green basil varieties might contain elevated amounts of these compounds, potentially enhancing their antioxidant properties [38,50]. The presence of amino acids may enhance this antioxidant activity by providing the necessary building blocks for phenolic synthesis, thereby improving the plant’s resilience to high temperatures [51]. The application of L-Tryptophan or L-Phenylalanine had the highest antioxidant activity in “Dark Opal” and “Bordeaux”, while L-Isoleucine had the greatest positive effect in the green variety “Thai”.

In summary, high-temperature stress has a detrimental impact on *Sweet Basil’s* growth, chlorophyll content, phenolic synthesis, and antioxidant activity. However, foliar applications of amino acids L-Tryptophan, L-Glutamine, L-Methionine, L-Phenylalanine, and L-Isoleucine have been shown to alleviate stress caused by high temperatures. These amino acids contribute to improved nitrogen metabolism, chlorophyll preservation, phenolic compound production, and antioxidant activity. Collectively, these mechanisms enhance the plant’s ability to withstand heat stress. The findings of this research indicate that amino acid treatments may offer practical benefits in promoting *Sweet Basil’s* growth and quality under adverse environmental conditions. Our results clearly indicate that the application of amino acids in *Sweet Basil* may be useful for an environmentally friendly agricultural strategy.

## 4. Materials and Methods

### 4.1. Experiment Conditions

**Figure 7:**
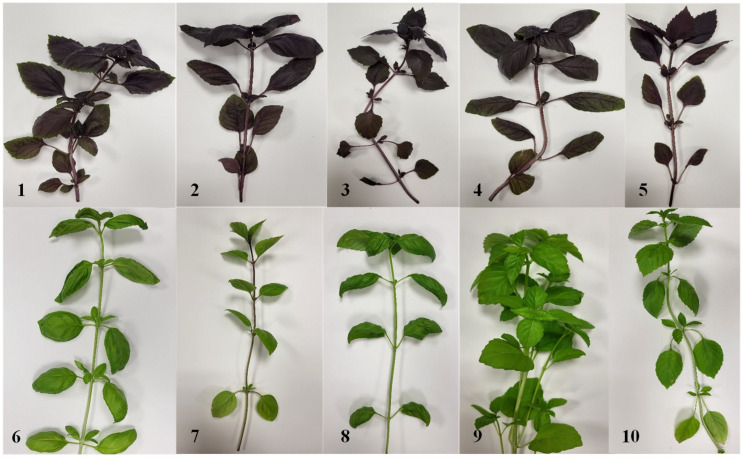
Varieties of purple *Sweet Basil* plants: (**1**)—“Rosie”; (**2**)—“Red Opal”; (**3**)—“Bordeaux”; (**4**)—“Dark Opal”; (**5**)—“Red Rubin”; varieties of green *Sweet Basil* plants: (**6**)—“Genovese”; (**7**)—“Cinamon”; (**8**)—“Italiano Classico”; (**9**)—“Marseillais”; (**10**)—“Thai”.

The experiment was performed at the open-access Joint Research Centre of Agriculture and Forestry, Vytautas Magnus University Agriculture Academy, in 2023 with ten cultivars of *Sweet Basil*: “Rosie”, “Red Opal”, “Bordeaux”, “Dark Opal”, “Red Rubin”, “Genovese”, “Cinamon”, “Italiano Classico”, “Marseillais”, and “Thai” (Figure 7).

The substrate for sowing was composed of raised bog peat and sand at a ratio of 3:1. The seeds were sown in plastic pots with a diameter of 12 cm and a height of 10 cm. Plants were grown in controlled-environment growth chambers with a 16:8 h photoperiod, 25/22 °C (day/night) temperature, and 150 µmol m^−2^ s^−1^ light density. Plants were thinned out to five per pot when the seedlings reached the cotyledon stage. The seedlings with 5–6 true leaves were divided into 7 groups: the first group of seedlings had no treatment and was grown under 25/22 °C (day/night) temperature, the second group of seedlings had no treatment and was grown under 35/30 °C (day/night) temperature, the third group of seedlings was sprayed with 100 mg L^−1^ of L-Isoleucine, the fourth group of seedlings was sprayed with 100 mg L^−1^ of L-Methionine, the fifth group of seedlings was sprayed with 100 mg L^−1^ of L-Glutamine, the sixth group of seedlings was sprayed with 100 mg L^−1^ of L-Tryptophan, and the seventh group of seedlings was sprayed with 100 mg L^−1^ of L-Phenylalanine. At 72 h after the application of the amino acids, when the plants were sprayed with a tested amino acid aqueous solution once (25 mL per 5 plants), the plants were subjected to a 35/30 °C (day/night) temperature (temperature stress), except the plants that were grown under 25/22 °C (day/night) temperature. Following a period of seven weeks from the initial stage of germination, the necessary samples for testing were collected. The experiment was performed in triplicate.

### 4.2. Determination of Chlorophyll Content

The contents of chlorophyll *a* and *b* were assessed following the methodology outlined by Sims and Gamon [52]. A total of 0.2 g of fresh, well-developed leaves were homogenized with acetone (80% *v*/*v*) and then centrifuged at 3000 rpm. The supernatant was used to measure absorbance at 663 and 647 nm using a spectrophotometer Spectro UV-VIS Dual-beam (Labomed Inc., Los Angeles, CA, USA).

### 4.3. Determination of Fresh and Dry Weights

Following the conclusion of the experiment, the entire plant material in each pot was excised at the soil surface level. The fresh weight of the shoots was then determined by means of a digital scale. Thereafter, the shoots were subjected to a drying process in a thermostat set at 55 °C, after which their dry weight was once more determined using the aforementioned digital scale. 

### 4.4. Determination of Phenolic Compound Contents and ABTS Radical Scavenging Capacity

The radical scavenging capacity of the phenolic compound content was determined in 96% ethanolic extracts, which were prepared by homogenizing 0.3 g of plant material in 10 mL of 96% ethanol solution. The homogenates were then subjected to a centrifugation process at 4500× *g* for a duration of 30 min, after which the resultant upper layer, termed the ’supernatant’, was utilized for further analysis. The Folin–Ciocalteu method was employed for the determination of the total phenolic compound content. A total of 300 µL of 0.2 M Folin–Ciocalteu reagent was added to a test tube containing 100 µL of plant extract and incubated for 10 min at room temperature in darkness. Subsequently, 5 mL of a 7.5% Na_2_CO_3_ solution was added, and the reaction mixture was left to incubate for a further 30 min at room temperature in darkness. The spectrophotometer (Spectro UV-VIS Dual-Beam, Labomed Inc., Los Angeles, CA, USA) was then used to measure the reaction’s optical density at a wavelength of 765 nm. To estimate the total phenolic content (TPC), a calibration curve was constructed using gallic acid solutions as standards. Samples were analyzed in triplicate, and the results were expressed as milligrams of gallic acid equivalent (GAE) per gram of dry weight. The total antioxidant activity of basil extracts was measured using ABTS+ (2,20-azino-bis (3-ethylbenzothiazoline-6-sulfonic acid) radical scavenging activity assays as described by Yim, Nam [53]. For analysis, 96% ethanolic extracts from ground basil leaves were used, and the antioxidant activity was expressed as mg Trolox equivalents (TE) per gram dry weight. The ABTS radical scavenging activity of the basil extracts was measured by the ABTS cation decolorization assay. The ABTS radical cation (ABTS+·) was produced by a reaction of 2 mM ABTS stock solution with 0.0095 g potassium persulfate, which was allowed to stand in the dark at room temperature for 16 h before use. Before analysis, the ABTS + solution was diluted with ethanol to give an absorbance of 0.8 ± 0.03 at 734 nm. For analysis, 20 µL of ethanolic basil extract was permitted to react with 3 mL of the ABTS+ solution for 60 min in the dark following the measurement of the mixture’s absorption at 734 nm. The radical scavenging capacity was expressed as the percentage of the inhibition of ABTS radicals using the following equation:

ABTS radical scavenging capacity (%) = (1 − (A_1_/A_0_)) × 100, (1)

where A_0_ is the absorption value of the ABTS itself, and A_1_ is the absorption value of the sample.

### 4.5. Statistical Analysis

The experiments were meticulously organized with complete randomization, and the assay was performed in triplicate. The data analysis was computed using the software package TIBCO Statistica, version 10 (TIBCO Software, Palo Alto, CA, USA). The statistical difference (*p* < 0.05) among the means was analyzed by Tukey’s post hoc test. The mean values of chlorophyll *a*, chlorophyll *b*, fresh weight, dry weight, phenolic compound contents, ABTS radical scavenging capacity, and standard error (SE) were calculated on the basis of the number of independent replicates. The effect of factors (amino acids, variety) and their interaction with investigated variables were studied by two-way ANOVA. The Tukey’s honestly significant difference (HSD) test was carried out with a significance level of *p* < 0.05.

## 5. Conclusions

The effects of exogenously applied amino acids L-Phenylalanine, L-Tryptophan, L-Glutamine, L-Methionine, and L-Isoleucine on the morphophysiological parameters, the total phenolic content (TPC), and the antioxidant activity of *Sweet Basil* under high-temperature stress were studied. The application of L-Tryptophan had the most effect on enhancing fresh and dry biomass in green *Sweet Basil* varieties, and L-Methionine had the most significant impact on biomass accumulation in purple *Sweet Basil* varieties. Application of L-Phenylalanine to high-temperature stressed plants significantly increased chlorophyll *a* and *b* contents in the purple variety “Bordeaux” and in the green variety *Sweet Basil* “Marseillais”. It was determined that L-Isoleucine and L-Glutamine had a significant influence on the increase in total phenolic compounds (TPCs) in the purple varieties’ cultivars “Rosie”, “Red Opal”, “Dark Opal”, and “Red Rubin”, while in the green varieties’ cultivars, the application of L-Tryptophan increased the TPCs in the cultivars “Cinamon” and “Italiano Classico” and the application of L-Phenylalanine increased the TPCs in the cultivars “Cinamon”, “Marseillais”, and “Thai”. It was determined that antioxidant activity measured by the ABTS method in purple *Sweet Basil* varieties grown under high-temperature conditions was the highest in “Dark Opal” and “Bordeaux” sprayed by L-Tryptophan or L-Phenylalanine. The greatest positive effect on antioxidant activity measured by the ABTS method in green *Sweet Basil* varieties grown under high-temperature conditions was determined in “Thai” sprayed by L-Isoleucine. In consideration of the results obtained from this study, foliar spraying with amino acids has been demonstrated as a practical approach to reducing the deleterious effects of temperature stress on *Sweet Basil*.

## Data Availability

Data are contained within the article.
